# Detecting the effects of predator-induced stress on the global metabolism of an ungulate prey using fecal metabolomic fingerprinting

**DOI:** 10.1038/s41598-021-85600-z

**Published:** 2021-03-17

**Authors:** Azzurra Valerio, C. Steven Borrego, Luigi Boitani, Luca Casadei, Alessandro Giuliani, Robert B. Wielgus, Stephanie L. Simek, Mariacristina Valerio

**Affiliations:** 1 School of the Environment, Washington State University, Pullman, WA USA; 2 Department of Biology and Biotechnologies, Sapienza University of Rome, Rome, Italy; 3 Department of Environment and Health, National Institute of Health, Rome, Italy; 4 Washington Department of Fish and Wildlife, Olympia, WA USA

**Keywords:** Animal physiology, Ecology, Metabolomics, Homeostasis, Metabolomics

## Abstract

Few field tests have assessed the effects of predator-induced stress on prey fitness, particularly in large carnivore-ungulate systems. Because traditional measures of stress present limitations when applied to free-ranging animals, new strategies and systemic methodologies are needed. Recent studies have shown that stress and anxiety related behaviors can influence the metabolic activity of the gut microbiome in mammal hosts, and these metabolic alterations may aid in identification of stress. In this study, we used NMR-based fecal metabolomic fingerprinting to compare the fecal metabolome, a functional readout of the gut microbiome, of cattle herds grazing in low vs. high wolf-impacted areas within three wolf pack territories. Additionally, we evaluated if other factors (e.g., cattle nutritional state, climate, landscape) besides wolf presence were related to the variation in cattle metabolism. By collecting longitudinal fecal samples from GPS-collared cattle, we found relevant metabolic differences between cattle herds in areas where the probability of wolf pack interaction was higher. Moreover, cattle distance to GPS-collared wolves was the factor most correlated with this difference in cattle metabolism, potentially reflecting the variation in wolf predation risk. We further validated our results through a regression model that reconstructed cattle distances to GPS-collared wolves based on the metabolic difference between cattle herds. Although further research is needed to explore if similar patterns also hold at a finer scale, our results suggests that fecal metabolomic fingerprinting is a promising tool for assessing the physiological responses of prey to predation risk. This novel approach will help improve our knowledge of the consequences of predators beyond the direct effect of predation.

## Introduction

Predators can affect prey demography and indirectly the dynamic of the whole ecosystem through the killing of prey (i.e., direct predation) and the cost of prey responses to the risk of predation (i.e., risk effects)^1–3^. While the relative strengths of these processes are heterogeneous and context-dependent^4^, measures of prey responses to risk have frequently been limited to estimating antipredator behavioral responses, such as alteration of habitat use, movement patterns, vigilance or grouping behavior^5–7^. However, we still know little about the physiological responses to predation risk and their effects on prey fitness^8^, particularly in large carnivore-ungulate systems (but see^9,10^). By altering biological functions (e.g., heart rate, metabolism, and immune system functions) stress response can influence growth, reproduction, and disease susceptibility in an animal under repeated exposure to environmental disturbances^11,12^. Therefore, due to the potentially deleterious effects on bodily functions, measures of stress response should be also considered when assessing the consequences of predators beyond the direct effect of predation.

Traditionally, single metrics such as hormonal and immunological changes, have been considered to be associated with physiological stress and therefore extensively used to measure the intensity of stimulation to which an animal is exposed^13–15^. However, the amplitude of the stress response is not only related to the type and intensity of the stressors, but it is also affected by the animal’s perception of that event^16–19^. Early experience, age, genetic predisposition, and social relationships are factors that can influence the way an animal perceives and reacts to a stressor and thus, results in interanimal variations in stress response^20–23^. Although in controlled settings it may be possible to account for the influence of these confounding effects, assessing the amplitude of stress response of animals in their natural environment remains challenging^24–26^. Therefore, as multifaceted interactions between genetic, metabolic and environmental factors are at the basis of stress response, there is a need to develop new strategies and systemic methodologies for stress detection of animals in their natural environment.

Over the past decade, research on the gut microbiome (the extensive variety of microorganisms that live in the gastrointestinal tract of mammal hosts) has shown that gut microbes sense emotional and cognitive alterations, and by interacting with the hypothalamic–pituitary–adrenal axis mediate the body’s response to stress^27–29^. While the precise mechanisms whereby the gut microbiome modulates stress response have not been clearly established (but see^30^), recent studies suggest that the temporal changes of the gut microbial metabolism in response to early life stress in chickens *Gallus gallus domesticus*^31^, mild-prolonged stress in mice *Mus musculus*^32^, and restraint and separation in cattle *Bos taurus*^33^ may aid in identification of stress. This detection function is possible because the gut microbial metabolism is directly linked to the host metabolism: low-molecular weight compounds (metabolites) produced by the host after metabolism and conjugation in the liver, are excreted in the intestine where they are further metabolized by the gut microbes^34^. Therefore, as the host metabolism is able to respond to stimuli within seconds^35,36^, the rapid metabolic changes that occur in an organism in response to external stressors are integrated into the host and gut microbial co-metabolism and can be detected in the feces^33^. The recent development of high-throughput techniques such as fecal metabolomics have become increasingly popular to non-invasively detect these subtle phenotypic changes^37^.

Metabolomic analysis applied to feces allows the determination of the fecal metabolome, which represents the concentration of unabsorbed metabolites coproduced by the host and its gut microbiome. Because each fecal metabolome (extracted from a sample collected at a specific time and under a well-defined state) has a characteristic metabolic composition, its profile can be used as a ‘signature’ or ‘fingerprint’ to detect any shift in the animal’s metabolism. Specifically, metabolomic experiments that involve the fingerprinting approach are designed to reveal the differences between metabolite profiles of control and test groups^38–41^; this approach does not attempt to identify all the metabolites, but rather to quantify a global measure of metabolism (fingerprint pattern) in relation to environmental stressors^42^. In this manner, the fingerprinting approach could be used in ecological studies as a rapid, diagnostic tool to detect the endogenous changes that occur in prey in response to predator presence^33^. For example, it can be used to compare the fecal metabolome of samples collected in areas with different predation risk across the sampling space (e.g., predator presence-absence, near-far from predator) and time (e.g., before-after predator reintroduction or removal) or at a finer spatial–temporal scale to monitor the alterations of the fecal metabolome in reaction to a specific situation (e.g., predator encounter).

Proton nuclear magnetic resonance (^1^H-NMR) spectroscopy and mass spectroscopy (MS) are the most common analytical techniques used to generate metabolomic data for gut microbiome studies^43^. Between the two techniques, MS is more sensitive in detecting a broader range of metabolites and possible biomarkers of stress^44^. However, ^1^H-NMR spectroscopy is a rapid tool, more reproducible than MS^38,45^ and thus, ideal for monitoring the response to stress using serial sampling of the same individual^33^. Moreover, because ^1^H-NMR signals are directly and linearly correlated to metabolite abundance, this technique provides a robust and unbiased measurement of the global metabolic state of an organism^38,46^.

In this study, we showed that fecal metabolomic fingerprinting can be used as a novel, non-invasive approach to investigate the physiological responses of a prey (cattle) induced by the presence of a predator (wolf *Canis lupus*) in a multiuse landscape of northeastern Washington (WA), USA. After 70 years of absence, wolves have recently begun recolonizing their historical range in WA where cattle are abundant and graze mostly untended on a mix of forested and semi-agricultural lands^47^. For three summer grazing seasons (2014–2016) we measured spatial overlap between wolves and range cattle using GPS radio-collars. By collecting longitudinal fecal samples from GPS-collared cows, we used ^1^H-NMR spectroscopy to contrast the fecal metabolome of cattle herds grazing in low vs. high wolf-impacted areas within three wolf pack territories. We then evaluated the importance of cattle distance to GPS-collared wolves, nutritional state of cattle, and other environmental variables (e.g., landscape and climate) to provide a mechanistic interpretation for the potential metabolic variability between herds. As predators are a major pressure for prey species^48^, we expected that the fecal metabolome of cattle herds would reflect the variations in wolf predation risk, and that distance to GPS-collared wolves would be one of the main factors associated with differences in cattle metabolism. Finally, to validate our results, we verified if the metabolic difference between herds in low and high wolf-impacted areas is a predictor of cattle distance to wolves.

### Study area

**Figure 1:**
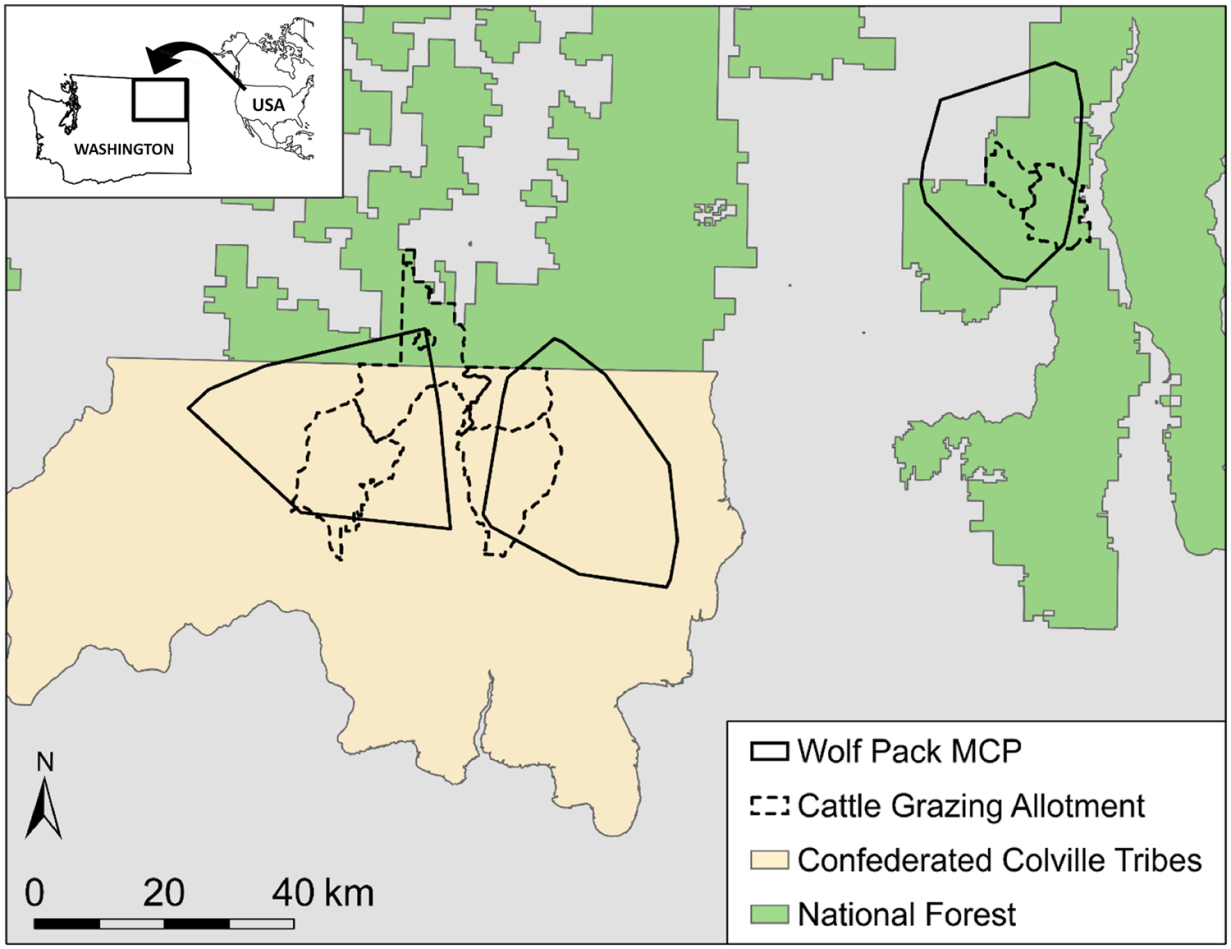
Study area in Northeastern Washington, USA, with minimum convex polygon (MCP) of wolf packs (n = 3) and cattle grazing allotments (n = 6, two allotments per pack) where wolf-cattle interactions were studied in 2014–2016. Map was created in ArcGIS 10.6 (ESRI, Redlands, CA) using data collected during the study and shapefiles provided by the Colville Tribes Fish and Wildlife Department, and Colville National Forest U.S.F.S.

Our study was conducted for three consecutive grazing seasons (2014–2016) in 4000 km^2^ of northeastern WA that included parts of the Colville Confederated Tribal Reservation and the Colville National Forest (Fig. 1). Towards the east, the area encompassed the foothills of the Selkirk Mountains characterized by forested lands interspersed with meadows and agricultural fields in the valley bottoms. The western portion of the study area included part of the Okanogan Highlands and Kettle River Range characterized by more rugged topography, and forested lands that transition southward into shrub lands and flat prairies. Elevations ranged from 290 to 2676 m, and the average temperature and precipitation across the grazing seasons were 17 °C (range: 8–26 °C) and 2.9 cm (range: 2.1–3.7 cm) respectively. The study area is used for multiple human activities including cattle ranching, which is practiced on public, private, and tribal lands. All the cattle herds in our study were beef cow-calf operations, composed primarily of adult Angus cows and a fewer number of heifers and breeding bulls. Typically, cattle are kept in smaller private pastures during the calving season (Nov-April), and then released with their calves to graze untended in forest and range allotments (mean size: 68.4 ± 52.2 km^2^) throughout the grazing season (May–October). Wild ungulates in the area, in addition to cattle, included white-tailed deer (*Odocoileus virginianus*), mule deer (*Odocoileus hemionus*), Rocky Mountain elk (*Cervus elaphus nelsoni*), and moose (*Alces alces*). Carnivores present in the study area were black bears (*Ursus americanus*), cougars (*Puma concolor*) and coyote (*Canis latrans*), although wolves were the predominant predator of cattle^49^.

## Methods

### Study design

We investigated the effects of wolf presence on cattle metabolism at three study sites (A, B and C), each containing a single wolf pack territory that overlapped the grazing allotments of two spatially separated cattle herds (Fig. 1). Because the metabolism is highly responsive to environmental changes, we considered each site as an independent study to minimize the site-specific effects. In each site, we used an integrated approach that consisted of two steps.

*Step 1.* We used metabolomic fingerprinting to compare the metabolome of fecal samples collected from GPS-collared cows in two spatially separated cattle herds grazing in the low and high wolf-impacted areas of the same wolf pack territory. To categorize the two herds into higher or lower-impacted wolf areas, we calculated utilization distribution overlap index (UDOI)^50,51^ between each herd and the wolf pack that overlapped their respective grazing area. The UDOI represented an index of spatial interaction and was based on the product of two utilization distributions calculated by kernel density estimators (for details see Supplementary Information). The UDOI values, that range between 0 (no overlap) and 1 (identical utilization distributions), were used to assign the two herds to either a lower or higher-impacted wolf area. The lowest value of UDOI indicated a lower-impacted wolf area, whereas the highest value of UDOI indicated a higher-impacted wolf area (Table 1). We used this conservative approach because monitoring a separate cattle herd farther from the wolf pack minimized the possible effect of non-collared pack members on the cattle metabolic shifts. Moreover, this design had the advantage of using a common treatment (i.e., wolf pack) in both lower and higher wolf areas.

**Table 1:** Sites with wolf packs, cattle herds, and utilization distribution overlap index (UDOI).

	Wolf pack			Cattle herd			UDOI	Year
	Name	Size	UD	Name	Size	UD	LW/HW	
**Site A**	Strawberry	8	145 km^2^	Herd-A1	105	57 km^2^	0.21 HW	2014–2015*
				Herd-A2	120	90 km^2^	0.01 LW	
**Site B**	Smackout	12	156 km^2^	Herd-B1	85	36 km^2^	0.26 HW	2016
				Herd-B2	105	45 km^2^	0.05 LW	
**Site C**	Nc’icn	7	345 km^2^	Herd-C1	90	99 km^2^	0.05 HW	2016
				Herd-C2	110	41 km^2^	0.00 LW	

Wolf pack size is the minimum number of wolves which included adults, juveniles, and pups of the year, and was determined using remote trail-cameras and direct pup counts from May to October. Cattle herd size was the number of cow-calf pairs. Utilization distributions (UD) were estimated by a 95% kernel density estimator using GPS points collected between May and October (see Supplementary Information). UDOI, which is based on the product of two utilization distributions (95% volume contour) (see Supplementary Information), represented an index of spatial interaction between wolves and cattle; within each site lower values of UDOI indicate herds in lower wolf-impacted area (LW), and higher values of UDOI indicate herds in higher wolf-impacted area (HW).

*Data for site A were combined and averaged for the years 2014 and 2015.

*Step 2.* To provide a mechanistic interpretation of the metabolic variability between the two herds, we assessed the importance of the following external variables on the cattle fecal metabolome: (a) *Distance to GPS-collared wolves.* Wolf presence, varying through space and time over the course of the grazing season, may affect the metabolic variability of both cattle herds at a finer spatial–temporal scale. Therefore, using GPS telemetry data we assigned to each fecal metabolome (obtained from a fecal sample) a measure of ‘wolf distance’ calculated in the 48 h prior to the deposit of the feces. We determined this time through a stress-induced test on cattle and found that the fecal metabolome can reflect short-term stress responses for an average of 48 h^33^. Although the duration of stress response can vary among individuals^33^ and different stressors, we assumed that if a cow perceived or interacted with wolves during this time frame, a metabolic response would be detected in their feces. Therefore, in our field study, with 'time 0' as the moment that the feces was voided, we subdivided the previous 48 h in 24 2-h periods (or 4 12-h periods, in site A) according to the GPS schedule of collared animals. For each of the 2-h periods (or 12-h periods, in site A) we calculated (ArcGIS 10.6, ESRI, Redlands, CA) the distance between the closest wolf-cow GPS point locations. The average of these 24 wolf-cow distances (or 4 wolf-cow distances, in site A) represented the measure of wolf distance that we assigned to each fecal metabolome. (b) *Environmental and landscape factors.* Environmental stressors such as extreme temperature, dampness, or wind can negatively affect cattle health and productivity^52^ and potentially metabolism. Therefore, for each fecal sample, we measured the average daily temperature and precipitation in the 48 h prior to defecation. We obtained daily weather indices from the U.S. National Oceanic and Atmospheric Administration, after matching the location of each sample with the closest weather station in ArcGIS. In addition, as topographical variation can influence livestock energy expenditure^53^ and potentially metabolism, we calculated the average terrain ruggedness and slope for each sample in the 48 h prior to defecation. Based on the cattle’s GPS schedule, we subdivided the 48 h in 24 2-h periods. For each 2-h period, we extracted from the corresponding GPS location the value of ruggedness and slope using a 10 m^2^ digital elevation model in ArcGIS; their averages represented the values of ruggedness and slope that we assigned to each fecal metabolome. (c) *Cattle nutritional state*. In cattle, when forage quality is low, ingestion is reduced^54,55^ and animals spend more time harvesting and digesting low-quality diets^56^, potentially influencing their metabolism. Accordingly, we measured DAPA (2,6-diaminopimelic acid), a unique amino acid residue of rumen bacterial fermentation that is not absorbed by the animal and passes out through the digestive process in the feces^57^. An animal in excellent conditions is expected to have higher value of DAPA in the feces than an animal on low quality feed or with limited feed available. Because the passage rate of cattle ranges between 1 and 3 days^58^ we assumed that the feces reflected the nutritional well-being of the animal in the previous days. Therefore, we collected a subsample from the same feces used for metabolomics. Analyses were performed by the Wildlife Habitat and Nutrition Laboratory at Washington State University (WSU, Pullman)^57^.

### Data collection

#### Wolf and cattle telemetry data

Between 2014 and 2016, we outfitted GPS-collars (Vectronic Aerospace GmbH Inc. Berlin, Germany; Telonics Inc. Mesa, AZ, USA) on eight wolves in three wolf packs, and 65 cows in six livestock herds. Wolves were captured using either modified rubber-jawed foot-hold traps in summer, or by aerial darting in winter. At least one GPS collar was maintained in each pack to monitor wolf presence and movements during the grazing season. In each herd, GPS collars were outfitted on a random selection of matriarch cows (identified by livestock owners) as they tend to lead sub-herds or ‘groups’ of subordinates and juveniles, thereby influencing the behavior and habitat selection of the entire group^59^. Wolf and cattle locations were recorded every 2 h, except for site A in 2015 where the only GPS collar in the wolf pack was programmed to record a point location every 12 h. Because in site A the wolf GPS schedule differed between years, telemetry data were subsampled to a 12 h fix rate to use the same fix frequency in our analyses. A total of 7871 and 117,236 locations were recorded for wolves and cattle, respectively. To maximize the amount of information and minimize the effect of location error on spatial analyses, we retained all locations with a three-dimensional fix and excluded locations that had a two-dimensional fix if the dilution of precision was > 5^60^.

#### Cattle fecal collection

Fecal samples were randomly collected from GPS-collared cows every two weeks to distribute the collection across time and space; this best represented the spectrum of wolf effects on cattle metabolism over the grazing period. To collect fecal samples, we began by downloading GPS locations of collared cows. Once in the field, we used ground-based VHF radiotelemetry to locate and sight the cow. After the target animal was located, we observed cows maintaining a safe distance to reduce the likelihood of study-induced disturbances. When the feces were voided, we collected samples only if the animals moved away, or if they ignored our presence. Because bacterial action continues after the excretion of feces, we collected the samples as soon as possible (between 5 and 30 min) to preserve the integrity of the intestinal bacterial flora^61^. To avoid contamination, only the portion of the feces that did not contact the ground was collected using a sterile spatula. Samples were individually collected in cryovials (2 mL), homogenized, and stored temporarily in a dry shipper (CX100 Vapor Cryogenic Shipper, Taylor-Wharton) with liquid nitrogen, before being transferred to a − 80 °C freezer.

### Fecal metabolomic analysis

**Figure 2:**
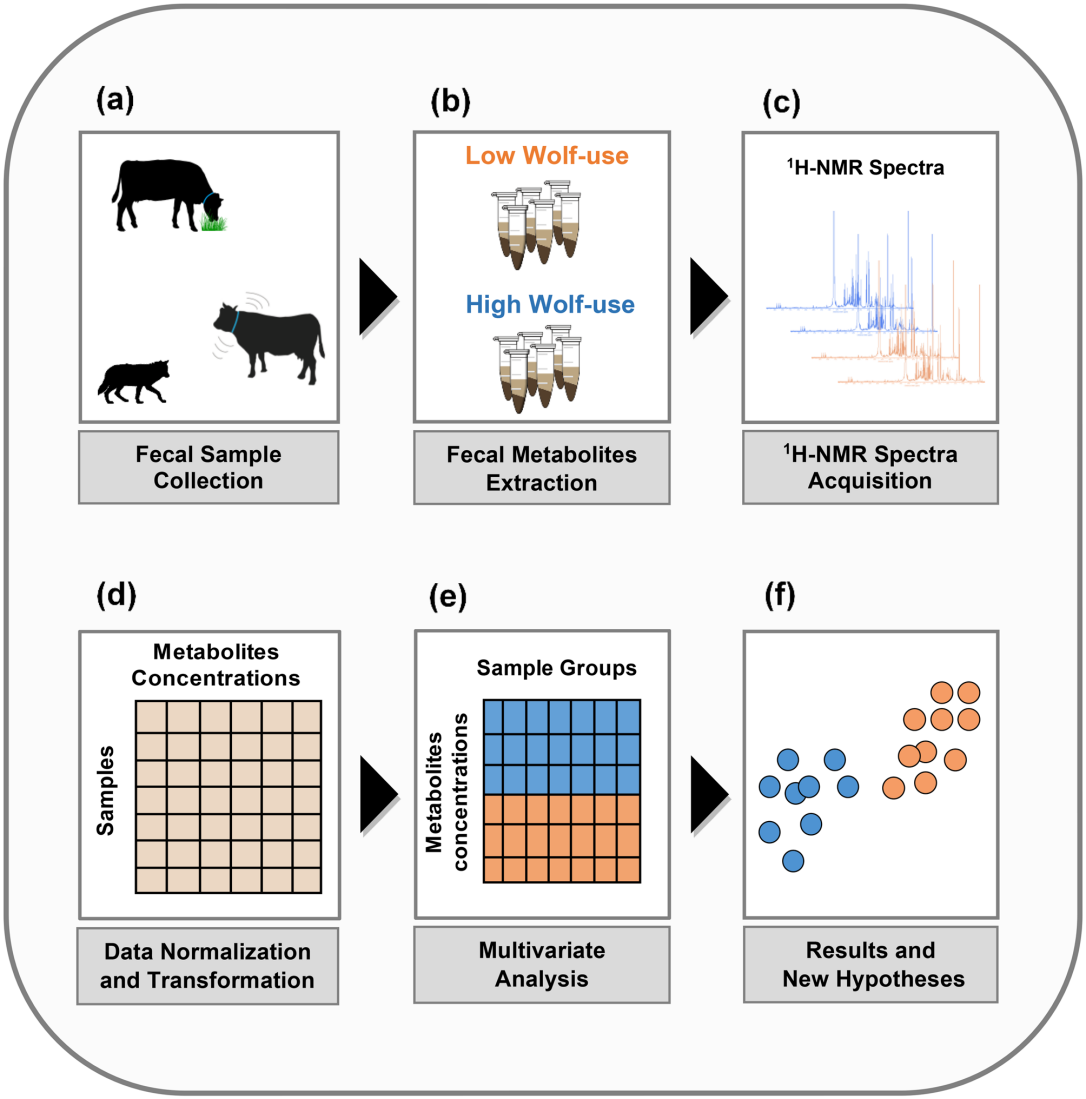
Steps in the fecal metabolomic analysis (**a**–**f**). Figure was created in Abode Illustrator CC (2015).

^1^H-NMR spectroscopy applied on a fecal extract allows the simultaneous detection of various metabolites of interest (e.g., amino acids, organic acids, carbohydrates, and short-chain fatty acids). Because the ^1^H-NMR spectrum of the whole fecal metabolome is extremely complex due to the presence of hundreds of low-molecular weight compounds, multivariate analysis is an integrated part of metabolomics providing simplified models for highly inter-correlated data^62^ (Fig. 2). We performed the ^1^H-NMR sample preparation, spectra acquisition and spectra processing treatment according to procedures previously described in Valerio et al.^33^.

#### ^1^H-NMR sample preparation

Feces were briefly thawed, and approximately 200 mg were transferred into a sterile tube with 6 mL of water (see Supplementary Information). Samples were vortexed for 2 min and centrifuged at 14,000 *g* for 10 min at 20 °C. From the fecal water, 4 mL of supernatant was collected and frozen at − 80 °C. Subsequently, frozen samples were freeze dried for 48 h. The resulting powder was dissolved in 1 mL of 10 mM deuterium oxide (D_2_O) (99.9 atom% D) phosphate buffered saline solution at pH = 7.4 containing sodium salt of 1 mM 3-(trimethylsilyl) propionic-2,2,3,3-d_4_ acid (98 atom % D) (TSP) and 10 mM sodium azide (NaN_3_). After centrifugation, 800 μL of each resulting supernatant was transferred into a 5 mm NMR tube.

#### ^1^H-NMR spectra acquisition

After the preparation of fecal water samples, the ^1^H-NMR spectra were acquired on a Varian 400 MHz Spectrometer at the Center for NMR Spectroscopy (WSU, Pullman) using a CPMG (Carr-Purcell-Meiboom-Gill) presat pulse sequence with an inter-pulse delay (τ) of 1 ms and a big-tau (180° refocusing pulses) of 0.6 s. The 90° pulse length was 7.16 μs. A total of 64 scans were collected into 8 k data points with a spectral width of 6410.26 Hz ppm.

#### ^1^H-NMR spectra processing treatment

The free induction decay (FID) signals were zero-filled to 64 k data points and multiplied by an exponential function with a line-broadening factor of 0.3 Hz before Fourier transformation. ^1^H-NMR spectra were manually corrected for phase and baseline distortions and calibrated to the TSP signal at *δ* 0.0 ppm. Then, the spectra were reduced into spectral bins with widths ranging from 0.01 to 0.03 ppm by using the ACD intelligent bucketing method (1D NMR Manager software (ACD/Labs, Toronto, Canada). Binning was performed excluding the residual water region (δ 4.66–4.80 ppm). The resulting bins were integrated and normalized with respect to the total integral region to generate the data matrix for multivariate analysis.

#### Multivariate pattern recognition analysis

SIMCA-P + v.13.0.3 (Umetrics AB, Umeå, Sweden) was used to perform principal component analysis (PCA) and orthogonal projection to latent structure discriminant analysis (OPLS-DA). For each site, the unsupervised method of PCA was initially applied to all the ^1^H-NMR spectra (obtained from the feces collected in lower and higher wolf areas) to find hidden metabolic patterns and group data without any prior information on the identity of the samples^62^. OPLS-DA, a supervised method in which the identity of samples is included in the model, was subsequently applied to maximize the separation between the two groups and to obtain a better prediction of the variables. Contrary to PCA, OPLS-DA separates the systematic variation in the matrix X (^1^H-NMR spectra) into one predictive component, or latent variable (LV), that is linearly related to the matrix Y (studied groups) and a single or multiple LVs orthogonal to the matrix Y that do not contribute to the discrimination of the studied groups^41^. This partitioning of the X data into predictive and orthogonal LVs, while blurring the original latent structure, improves the interpretation of the model^63^. The output from PCA and OPLS-DA consists of score plots, which provide a visual indication of the differences between the two groups in terms of metabolic similarity and loading plots (i.e., the correlation coefficients between original variables and components). Model fitting and predictive ability were assessed using the parameters R^2^X, R^2^Y and Q^2^. T-tests were used in a complementary manner to assess the significance of the separation between groups in PCA and OPLS-DA score plots. To further explore the results obtained by OPLS-DA, we identified the ^1^H-NMR variables (i.e., metabolites) that were significant for the discrimination of the studied groups. The first step was to select the spectral bins with the variable importance in the projection score (VIP) > 0.9 and the *P*-value < 0.05 estimated by 95% Hotelling’s T^2^ ellipse. Subsequently, metabolites were identified using Chenomx NMR Suite 7.72 (Chenomx Inc, Edmonton, Canada), an in-house NMR database, and literature data^64^; the assignment of metabolites was confirmed by 2D homo- and heteronuclear NMR spectroscopy experiments.

#### Spearman’s correlation analysis

To provide a mechanistic interpretation of the metabolic differences between the two herds within each site, we investigated the correlations between the OPLS-DA scores (LV1) and several external variables (described above) using Spearman’s correlation analysis. External variables were previously correlated in pairs to identify collinearity issues (|r|> 0.6). This filtering procedure excluded only the slope variable as highly correlated with ruggedness in all three sites.

#### Multiple regression analysis

To validate our results, for each site we verified if the potential metabolic difference between herds in lower and higher wolf-impacted areas can predict wolf presence. Accordingly, we derived a multiple regression model using the known distance to GPS-collared wolves as dependent variable and the PCA scores extracted from the original database as predictors. Because the PCs are orthogonal to each other by construction, multicollinearity issue between the predictors was excluded a priori, and only the PCs most correlated with distance to wolves (Table S1) were used for the regression analysis. Finally, the goodness of fit of each model (normality of errors, homoscedasticity, and independence) was checked using the appropriate diagnostic tests and through visual inspection of the residuals.

### Ethical approval

All experimental protocols and data collection were approved by the WSU Institutional Animal Care and Use Committee (WSU IACUC: 04687-001; 04661-002). All experiments and methods were performed in accordance with relevant guidelines and regulations.

## Results

In site A, we collected a total of 190 cattle fecal samples during two consecutive grazing seasons (2014–2015). Sites B and C produced data for one grazing season (2016) during which we collected 90, and 65 fecal samples, respectively. We used each fecal sample to extract fecal metabolites and obtain ^1^H-NMR spectra (see Fig. S1 for an example of cow ^1^H-NMR spectrum).

### Fecal metabolome differs between cattle herds in lower and higher wolf-impacted areas

**Figure 3:**
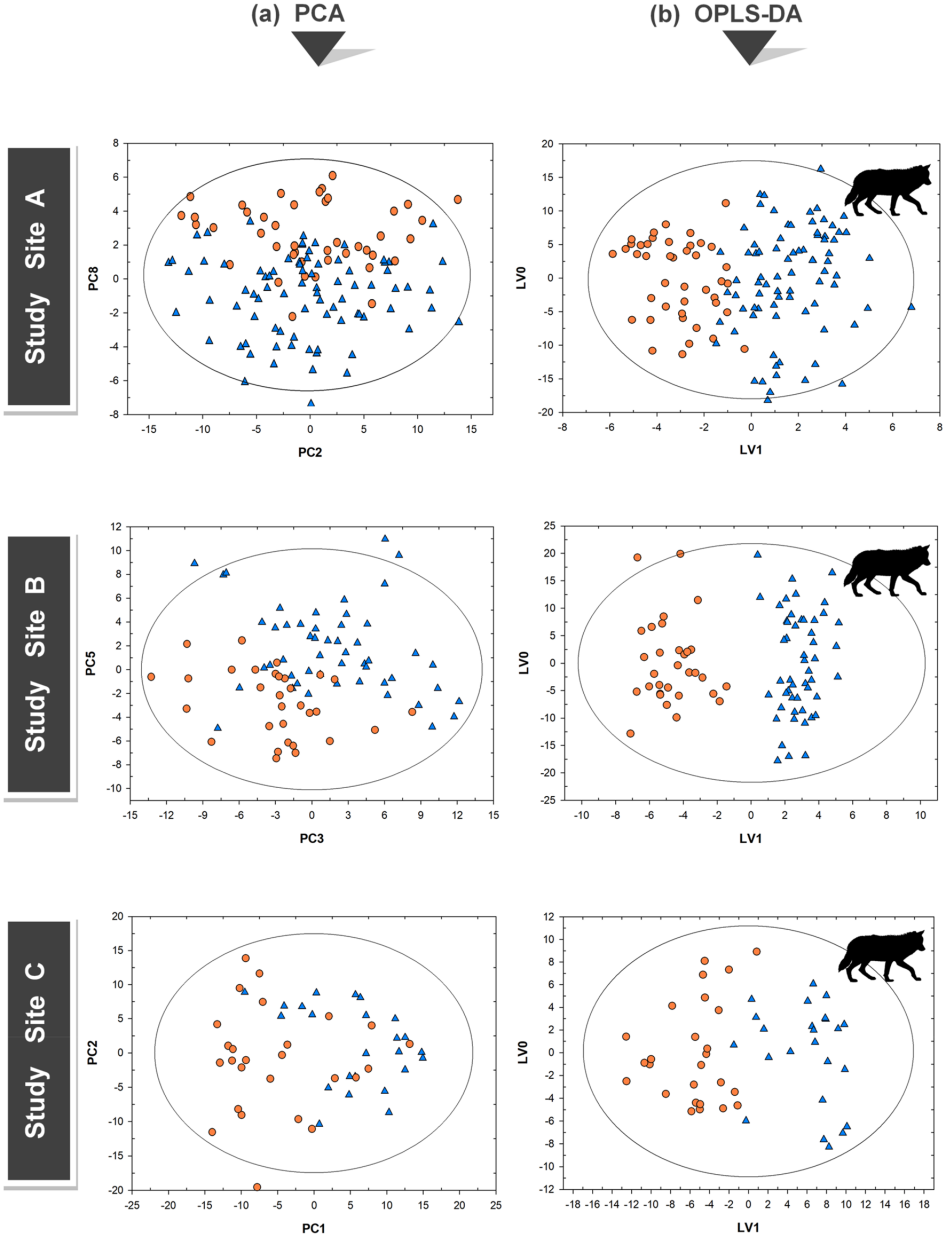
(**a**,**b**). Metabolic differences of cow fecal extracts in the three sites. (**a**) PCA score plots of ^1^H-NMR spectra of cow fecal extracts obtained from samples collected in lower (orange circles) and higher (blue triangle) wolf-impacted areas within each site. The differences between the specific pairs of fecal extracts were statistically significant: in site A on PC2 (*P* = 0.024), PC4 (*P* = 0.030) and PC8 (*P* < 0.0001); in site B on PC3 (*P* < 0.0001), PC5 (*P* < 0.0001), PC7 (*P* = 0.037) and PC8 (*P* = 0.031); in site C on PC1 (*P* < 0.0001). (**b**) OPLS-DA score plots showing the maximized discrimination between the ^1^H-NMR spectra of cow fecal extracts obtained from samples collected in lower (orange circles) and higher (blue circles) wolf-impacted areas within each site. The differences between the specific pairs of fecal extracts were statistically significant (*t*-test, *P* < 0.00001) in all three sites. Plots were produced in SIMCA-P + v.13.0.3 (Umetrics AB, Umeå, Sweden). Abode Illustrator CC (2015) was used to combine the plots into one figure and to create the wolf drawing.

For each site, we initially performed the unsupervised method of PCA on all ^1^H-NMR spectra obtained from the two cattle herds in lower and higher wolf-impacted areas of the same pack territory (Fig. 3a). For site A, the PCA produced a solution with 12 significant components, cumulatively explaining 84% of the total variance in the data (Q^2^ = 0.71). The PCA for site B produced a solution with nine significant components, cumulatively explaining 76% of the total variability (Q^2^ = 0.63). Lastly, the PCA for site C produced a solution with six significant components, cumulatively explaining 55% of the total variance in the data (Q^2^ = 0.33). We found significant differences (*t*-test) between all the specific pairs of cattle herds: in site A on PC2 (*P* = 0.022), PC4 (*P* = 0.030) and PC8 (*P* < 0.0001); in site B on PC3 (*P* < 0.0001), PC5 (*P* < 0.0001), PC7 (*P* = 0.037) and PC8 (*P* = 0.031); in site C on PC1 (*P* < 0.0001).

After observing a natural (unsupervised) clustering of the fecal metabolic profiles with PCA, we analyzed the same ^1^H-NMR datasets using the supervised method of OPLS-DA (Fig. 3b). Considering two groups of ^1^H-NMR spectra (obtained from cattle herds in lower and higher wolf-impacted areas), we obtained: in site A, a good predictive model (Q^2^ = 0.53) with one predictive and three orthogonal LVs with R^2^X = 55% and R^2^Y = 66%; in site B, a good predictive model (Q^2^ = 0.73) with one predictive and three orthogonal LVs showing R^2^X = 51% and R^2^Y = 90%; and in site C, a predictive model (Q^2^ = 0.35) with one predictive and one orthogonal LVs showing R^2^X = 27% and R^2^Y = 74%. *T*-tests applied on the predictive LV1s confirmed the significant differences between the specific pairs of cattle herds in all sites (*P* < 0.00001). For each OPLS-DA model we identified the metabolites with a VIP score > 0.9 that significantly contributed to the discrimination between the two cattle herds in low and high wolf-impacted areas. A total of 20 metabolites were obtained from the OPLS-DA model of site A; 21 metabolites were obtained from the OPLS-DA model of site B, and 17 metabolites from the OPLS-DA model of site C (Figure S2). These metabolites included amino acids and analogues, carbohydrates, carboxylic acids and derivatives, fatty acids, organonitrogen compounds, phenylpropanoic acids, and phenylacetic acids (Figure S2).

### Variables associated with differences in cattle metabolism

To provide a mechanistic interpretation of the metabolic difference between the two herds within each site, we correlated the OPLS-DA scores (LV1s) with several external variables. The Spearman’s correlation analysis showed that the metabolic variations on LV1s were mainly associated with distance to GPS-collared wolves explaining the clustering of metabolic profiles in two of three sites (site A, *r* =  − 0.55; site B, *r* = 0.73; site C, *r* = 0.29) (Table 2).

**Table 2:** Spearman’s simple correlation between the OPLS-DA scores of cow fecal extracts and external variables influencing the global metabolism of cattle.

	Wolf distance	Ruggedness	Temperature	Precipitation	DAPA
**Site A**					
r *p*-value	**0.55** 0.000	− 0.32 0.000	0.23 0.014	0.04 0.621	0.12 0.000
**Site B**					
r *p*-value	**0.73** 0.000	− **0.52** 0.000	0.04 0.622	− 0.04 0.616	− 0.15 0.194
**Site C**					
r *p*-value	− 0.29 0.152	− 0.42 0.003	− 0.34 0.014	0.23 0.121	− **0.51** 0.000

Correlation values |r|≥ 0.5 with *P-*value < 0.05 are highlighted in bold text. Wolf distance is defined as the average distance of the closest wolf-cow GPS points in the 48 h prior to the deposit of the cow feces; ruggedness is the average ruggedness calculated from cattle GPS points in the 48 h prior to defecation; temperature and precipitation are the average daily temperature and precipitation calculated in the 48 h prior to defecation; 2,6-diaminopimelic acid (DAPA), that measures nutritional quality, is the relative quantity of residue of rumen bacterial fermentation 1–3 days prior to defecation.

### Metabolic differences between cattle herds can predict wolf distance

**Figure 4:**
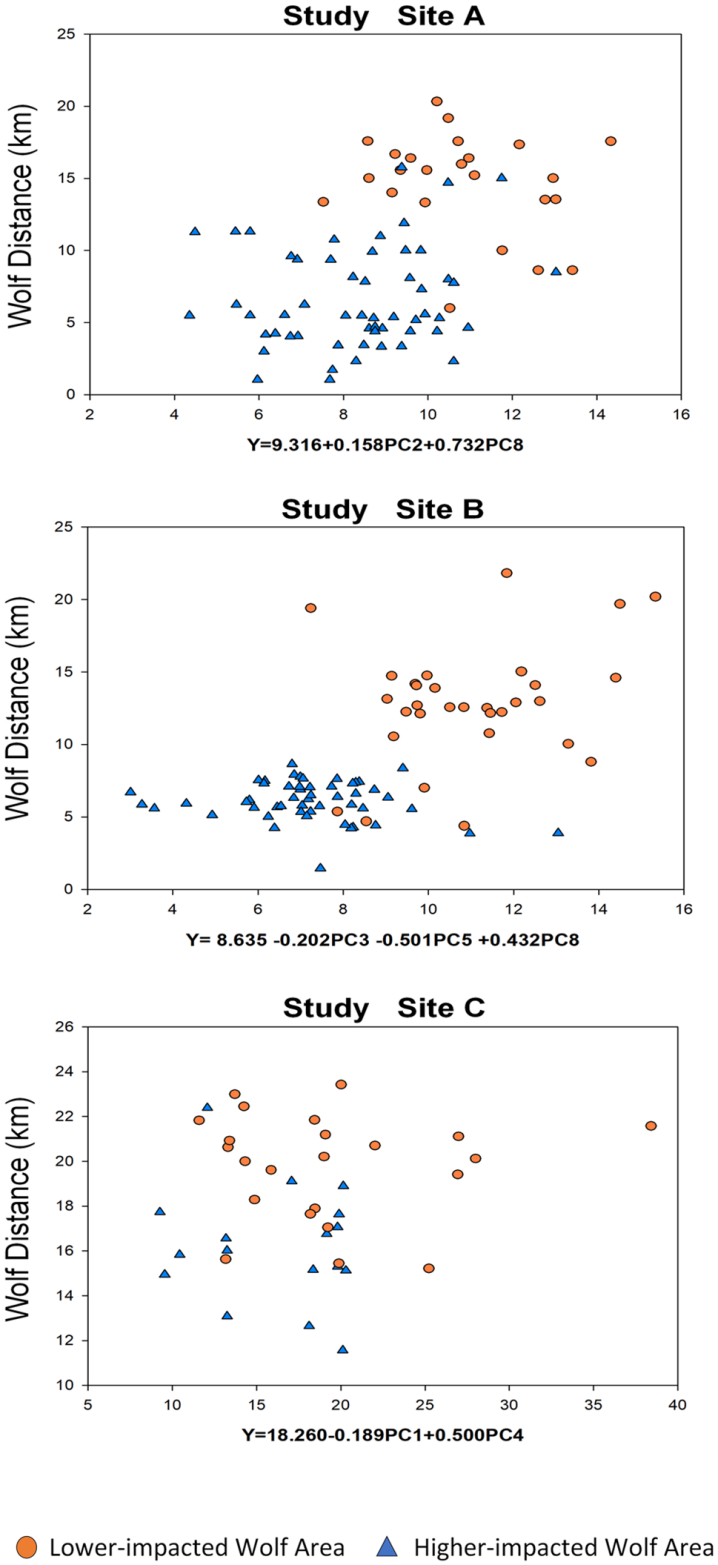
Relationship between cattle distance to GPS-collared wolves as estimated by a multiple regression model based on the PCs most correlated with distance to wolves and the observed distance values. Individual point distances are labelled with different colors based on the lower (orange circle) or higher (blue triangle) wolf-impacted areas where the distances were measured.

To validate our results, for each site we reconstructed the cattle distances to GPS-collared wolves by using the following multiple regression models based on the metabolic differences between the two herds (i.e., PCs most correlated with distance to wolves (Table S1)): site A, Y = 9.316 + 0.158PC2 + 0.732PC8, (*F* = 8.051; *P* = 0.001 and r = 0.42); site B, Y = 8.635 + (-0.202PC3) + (-0.501PC5) + 0.432PC8 (*F* = 15,249; *P* = 0.000 and r = 0.61); site C, Y = 18.260 + (-0.189PC1) + 0.500PC4 (*F* = 6.293; *P* = 0.004 and r = 0.49). Observed wolf distances, categorized in lower and higher wolf-impacted areas, were graphed against predicted values to illustrate the predictive ability of the regression models (Fig. 4).

## Discussion

In our study we used NMR-based fecal metabolomic fingerprinting as a novel non-invasive approach to detect the effects of predator-induced stress on the fecal metabolome of a prey (i.e., the responses of hundreds of metabolites coproduced by the host and its gut microbiome). Because the fecal metabolome maintains a ‘chemical memory’ of stressful events^33^, we were able to contrast the metabolite profiles of two spatially separated cattle herds in lower and higher wolf-impacted areas of the same wolf-pack territories. In the longitudinal fecal samples of GPS-collared cattle, we found relevant metabolic differences between the two herds in each of the three sites (Fig. 3). Although this metabolic difference was statistically significant with both PCA and OPLS-DA, the predictive ability of both models in site C (Q^2^ = 0.33; Q^2^ = 0.35) were relatively low compared to site A (Q^2^ = 0.71; Q^2^ = 0.53) and B (Q^2^ = 0.63; Q^2^ = 0.73). This result was confirmed by a further analysis on the most important metabolites that contributed to the discrimination between cattle herds in low and high wolf-impacted areas. From the OPLS-DA models of site A and B we obtained 16 shared metabolites, but of those only nine were also present in the OPLS-DA model of site C (Figure S2). It is possible that this difference occurred because in site C both cattle herds were separated from the wolves for most of the grazing season compared to the other sites (Table 1). In fact, starting from early summer, wolves denned outside both grazing allotments, and the probability of interaction with GPS-collared cattle was relatively low (Table 1). Interestingly, with this inadvertent design of ‘non-wolf’ and ‘low-impacted’ wolf areas, the metabolic variation between herds in site C could be related to other factors besides wolf presence. Therefore, to provide a better interpretation of these results, we further investigated whether distance to GPS-collared wolves, nutritional state of cattle, or aspects pertaining to other environmental perturbations (e.g., landscape and climate) could be related to the variation in cattle metabolism. Our results showed that in two of the three sites (A, and B), distance to GPS-collared wolves was the most correlated factor with differences in cattle metabolism of the parameters evaluated (Table 2). In site B, rugged terrain was also associated with differences in cattle metabolism, but at lower level than wolf distance (Table 2). In this case, separating the contribution of wolf proximity from topographical variation is challenging, as cows may have used steeper slopes and rugged terrain as refugia from wolves^65^. To confirm, we identified cattle selection for ruggedness in the high wolf-impacted area of site B^66^, which was also the area where the probability of interactions with wolves was higher compared to other sites (Table 1). Although distance to wolves resulted in the highest level of correlation with differences in cattle metabolism within sites A and B, we did not observe the same correlation pattern in site C, where both cattle herds were relatively distant from the wolf pack. Terrain ruggedness and cattle nutrition were the major factors associated with the variation in cattle metabolism at site C (Table 2), which could be related to differences in the characteristics of the two grazing allotments. The results of this correlation analysis confirmed what we partially observed in the first data-processing step and provided an additional, although purely mechanistic, interpretation for the metabolic variability between herds. However, that a lower wolf effect resulted in a lower metabolic response in site C was ultimately confirmed by a regression analysis used to reconstruct the distances to GPS-collared wolves based on the original (PCs) metabolic datasets. Our results showed that, contrary to sites A and B, the metabolic variability between herds in site C was not detailed enough to sort the estimated distances in lower and higher wolf areas; in fact, the two groups of distances are scattered along the regression line without any neat separation (Fig. 4). This additional data-processing step provided further evidence for the effect of wolf-induced stress on the fecal metabolome of range cattle, and indirectly for the discrimination ability of the fecal metabolomic fingerprinting approach.

Our results demonstrated relevant metabolic differences between cattle herds in areas where the probability of wolf pack interaction was higher (in site A and B). Moreover, although distance to GPS-collared wolves was the primary factor associated with this difference in cattle metabolism, the results of the correlation analysis might have been confounded by other factors that we were unable to measure or control for, but that can still influence the results. For example, cow temperament may be a potential factor that influences the prey’s reaction to a threat and consequently the metabolic shifts as a function of stress. Similarly, human presence is a factor that could be perceived by cattle as security from predators and thus influence the metabolic responses to wolves. Therefore, our analysis is only limited to the parameters evaluated, and distance to wolves could be differentiating the two herds together with an unknown number of other variables. Additionally, we observed patterns consistent with a wolf effect on cattle metabolism only at a large scale (e.g., between cattle herds). Considering that many responses to carnivores often occur at finer spatial and temporal scales^67^, further research is needed to explore if similar patterns also hold at a finer scale; this would provide additional support for the impact of wolves on the cattle fecal metabolome.

The NMR-based metabolomic fingerprinting approach that we proposed is a diagnostic tool widely used in medicine for sample classification, for instance to distinguish among samples with different metabolic state^40^. This approach does not attempt to identify all the metabolites but rather to characterize the global metabolic signature of the organism and to detect any shifts in its metabolism^42^. Because the fecal metabolome is highly sensitive to environmental influences and can change continuously, our intent was to get an unbiased global measure of metabolism without aiming to get quantitative data for all biochemical pathways. This is an inherent challenge particularly when measuring the metabolites of a free-living population, which would include considerable inter-individual metabolic variations^68^. Therefore, although the fingerprinting approach can provide evidence of stress on a comparative basis and does not provide a univocal marker of stress, we believe it can be an effective, rapid technique for the detection of stress in wildlife research^33^. In fact, because fecal metabolomics provides a final downstream product of the host-gut microbial activity at specific times, it offers a robust measurement of the interactions between the organism’s global metabolism and its surroundings.

In conclusion, we observed differences at a broader scale in the global metabolism (i.e., host-gut microbial co-metabolism) of range cattle in relation to the variation in wolf predation risk. These results are in line with recent studies on captive animals, indicating that stress and anxiety related behaviors alter the function of the gut microbiome and these metabolic alterations may aid in identification of stress^31–33^. The finding of patterns consistent with a wolf effect on the cattle global metabolism suggests that the alterations of the fecal metabolome may serve as a promising tool for detecting the physiological responses of prey to predation risk. In this sense, the NMR-based fecal metabolomic fingerprinting approach could be used to characterize the fecal metabolome of ungulates or other prey species, and its alterations related to predator presence both on a space and time scale. Our research represents a novel approach for detection of predator-induced stress, and its use, combined with other measures of prey responses to risk, will help improve our knowledge of the consequences of predators beyond the direct effect of predation.

## Supplementary Information

Supplementary Information

## Data Availability

The GPS data pertaining to the location of wolves and cattle used to generate the results of this study may be made available on a case-by-case basis in accordance with the institutions managing those GPS datasets. Cattle metabolomic data and external variables datasets may be made available on a case-by-case basis from the correspondent author.
