# Supplementary material for: Detecting the effects of predator-induced stress on the global metabolism of an ungulate prey using fecal metabolomic fingerprinting
Source: Sci Rep. 2021 Mar 17;11:6129. doi: 10.1038/s41598-021-85600-z (PMC7971053; doi:10.1038/s41598-021-85600-z)
Supplement: Supplementary file 1 — Supplementary Information [file 41598_2021_85600_MOESM1_ESM.pdf]

Supporting Information

Detecting the effects of predator-induced stress on the global metabolism of an ungulate prey using fecal metabolomic fingerprinting

Azzurra Valerio[*a], C. Steven Borrego [a], Luigi Boitani [b], Luca Casadei [a], Alessandro Giuliani [c], Robert B. Wielgus [a], Stephanie L. Simek [d] and Mariacristina Valerio [a]

[a] School of the Environment, Washington State University, Pullman, WA, USA

[b] Department of Life Sciences, University of La Sapienza, Rome, Italy

[c] Department of Environment and Health, National Institute of Health, Rome, Italy

[d] Washington Department of Fish and Wildlife, Olympia, WA, USA

*Corresponding Author: Azzurra Valerio

Email: azzurra.valerio@wsu.edu

Table of Contents

Methods. Utilization distribution overlap index. Pages S-3, S-4

Methods. ^{1}H-NMR sample preparation. Page S-5

Figure S1. Example of two ^{1}H-NMR spectra of cow fecal extracts obtained from samples collected in lower and higher wolf-impacted areas of Site A. Page S-6

Figure S2. VIP score plots of the most important metabolites that contributed to the discrimination of the two cattle herds in low and high wolf-impacted areas within site A, B, and C. A total of 20, 21, and 17 metabolites, with a cut-off value of VIP > 0.9 and *P*-value < 0.05, were obtained from the OPLS-DA model of site A, B and C, respectively. For site A and B, where the interactions with wolves were higher compared to site C (Table 1), we identified 16 shared metabolites: 3-hydroxyisovalerate, 3-phenylproprionate, alanine, butyrate, glucose, isobutyrate, lactose, N-acetylcysteine, N-nitrosodimethylamine, proprionate, sarcosine, succinate, Unknown 1 (aromatic region), Unknown 2 (aromatic region), valerate and valproate. Of these 16 metabolites, nine were also found in site C: alanine, butyrate, glucose, N-acetylcysteine, N-nitrosodimethylamine, proprionate, 3-phenylproprionate, valerate and valproate. Pages S-7, S-8

Table S1. Pearson's simple correlation between the PCA scores of cattle fecal extracts and distance to GPS radio-collared wolves for each site. Page S-9

Supplementary Information

Methods - Utilization distribution overlap index

In each study site, we used utilization distribution overlap index (UDOI) to quantify space-use sharing between each herd and the wolf pack that overlap their respective grazing areas. UDOI, which is based on the product of two utilization distributions, is a common metric used to assess home range overlap and is also a good proxy of encounter probability (Fieberg & Kochanny, 2005; Robert, Garant & Pelletier 2012). To calculate utilization distributions (UD) for both wolves and cattle, we used GPS telemetry data obtained from radio-collared animals. We combined the GPS telemetry data obtained from all radio-collared cows within each herd and from all radio-collared wolves within each wolf pack. If only one wolf was outfitted with a GPS radio-collar within a pack, the UD was calculated by using telemetry data obtained from that single animal. This happened in only two occasions, in 2015 within site A and in 2016 within site C. However, because the total number of adults in these packs was three and two respectively, we assumed that the data obtained from both radio-collared animals were representative of their respective pack's movements. UDs were estimated by a 95% kernel density estimator using GPS points collected between 1 May - 15 October; in site A, GPS points were pooled for the 2014 and 2015 seasons. We conducted the analysis in R v3.6.0 (R Development Core Team 2019) using the R functions from Fieberg (2014) that allowed calculation of home ranges with the 'plug-in' method for choosing smoothing parameters. The smoothing parameter (or bandwidth) influences the weight of each data point within the probability distribution function and is particularly critical in determining outer contours (home range estimate) (Gitzen et al. 2006, Horne et al. 2006). Because there is no consensus as to which bandwidth parameter to use, we plotted several curves and chose the estimate that was most in accordance with our prior knowledge of animal density (Kie et al. 2010), which was based on our daily monitoring activity. To monitor the presence and movements of our study animals throughout the grazing season, we used a combination of different techniques, such as remote cameras, tracks and sign searches, and VHF ground-based telemetry. Because the kernel smoothing estimates coincided approximately with the additional information that we collected via monitoring activities, we considered this method appropriate for our datasets.

LITERATURE CITED

Fieberg, J., & Kochanny, C. O. (2005). Quantification of home range overlap: the importance of the utilization distribution. *Journal of Wildlife Management*, **69**, 1346-1359.

Fieberg, J. R. (2014). Home range overlap indices implemented using kernel density estimators with plug-in smoothing parameters and Program R. Retrieved from the Data

Repository for the University of Minnesota.

Gitzen, R. A., Millspaugh, J. J., & Kernohan, B. J. (2006). Bandwidth selection for fixed-kernel analysis of animal utilisation distributions. *Journal of Wildlife Management*. 70, 1334-1344.

Horne, J. S. & Garton, E. O. (2006). Selecting the best home range model: An information-theoretic approach. *Ecology*, 87, 1146-1152.

Kie, J. G., Matthiopoulos, J., Fieberg, J., Powell, R. A., Cagnacci, F., Mitchell, M.S., et al. (2010). The home-range concept: are traditional estimators still relevant with modern telemetry technology? *Philos Trans R Soc B-Biological Sci*, 365, 2221-2231.

R Core Team (2019) R: A language and environment for statistical computing. R Foundation for Statistical Computing, Vienna, Austria. URL: https://www.R-project.org/.

Robert, K., Garant, D., & Pelletier, F. (2012). Keep in touch: Does spatial overlap correlate with contact rate frequency? *Journal of Wildlife Management*, **76**(8), 1670-1675.

Methods - ^{1}H-NMR sample preparation

Before performing the analysis, we conducted several trials to test the sensitivity of the 400 MHz spectrometer. First, we divided a single fecal sample into four aliquots, 50, 100, 150 and 200 mg, from which we extracted the fecal water samples. Subsequently, we compared the spectra obtained from each fecal water sample and based on the results we choose to use the 200 mg aliquot because produced spectra with the higher signal-to-noise ratio. Note, the fecal water samples were additionally concentrated to improve the sensitivity by using a freeze dryer (for 48 h) before acquiring the spectra. Moreover, we started our trials with at least 50 mg of feces based on a previous study where Casadei et al. (2018) compared the fecal metabolome of young patients with cystic fibrosis and healthy children (controls) using a 50 mg of feces and a 500 MHz NMR spectrometer to acquire spectra.

LITERATURE CITED

Casadei, L., Valerio, M., & Manetti, C. (2018). Metabolomics: Challenges and Opportunities in Systems Biology Studies. In: M. Bizzarri (Eds.) *Systems Biology. Methods in Molecular Biology*, vol 1702 (pp. 327-336). Humana Press, New York, NY.

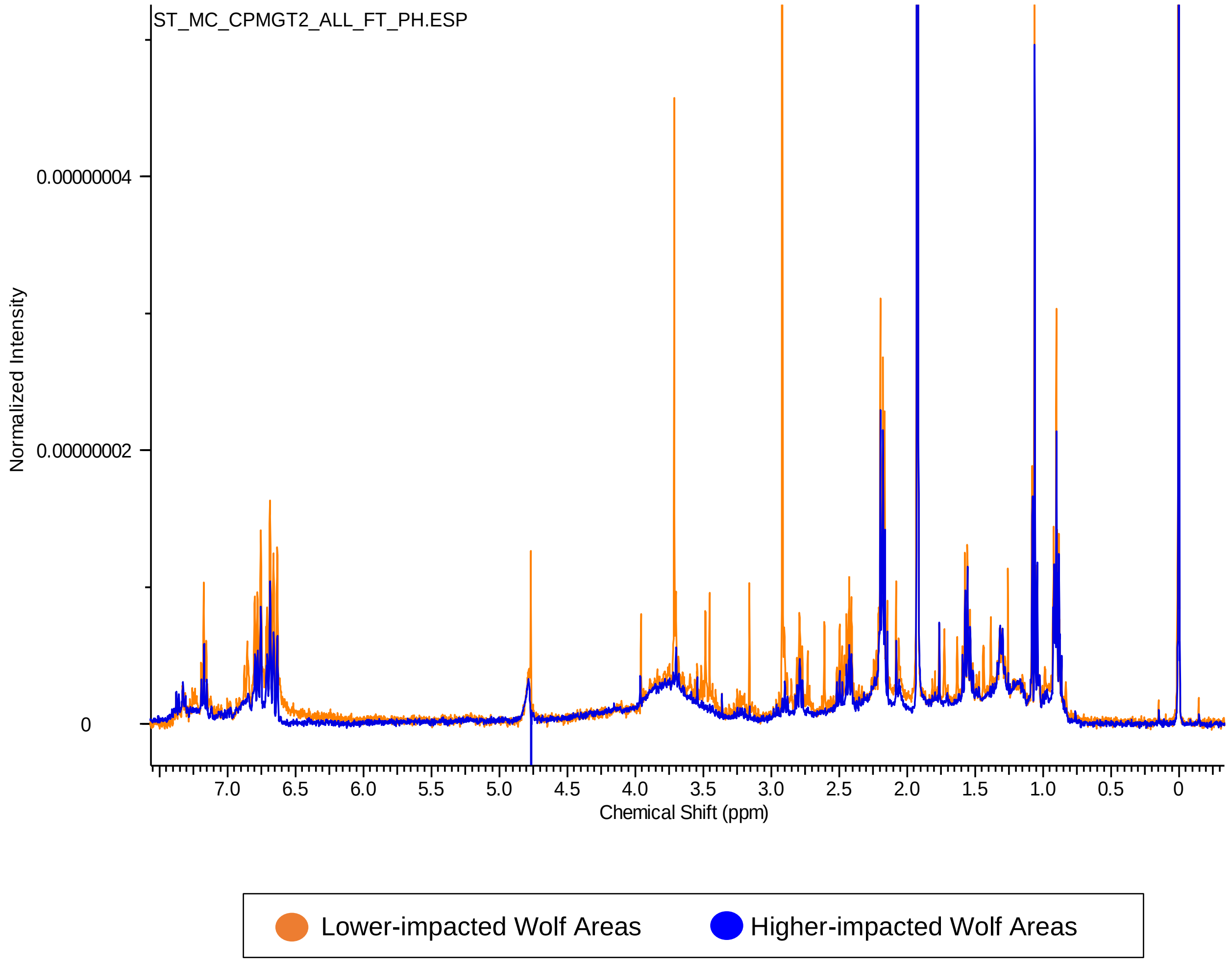

Figure S1. Example of two ^{1}H-NMR spectra of cow fecal extracts obtained from samples collected in lower (orange) and higher (blue) wolf-impacted areas of Site A.

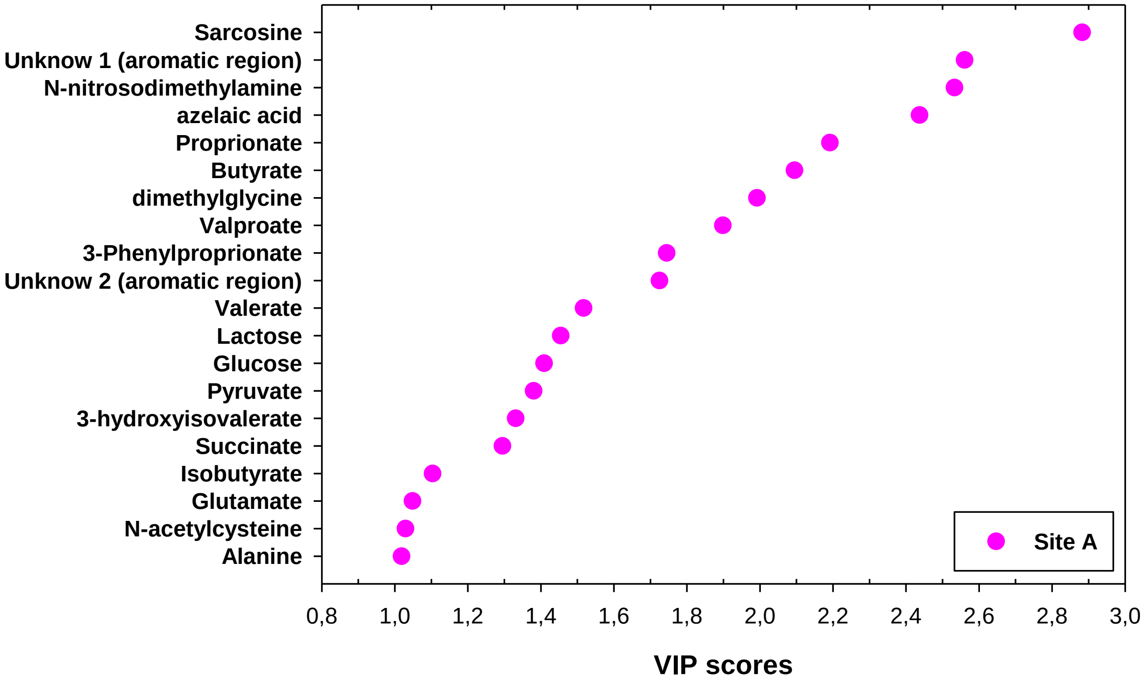
Sarcosine
Unknow 1 (aromatic region)
N-nitrosodimethylamine
azelaic acid
Proprionate
Butyrate
dimethylglycine
Valproate
3-Phenylproprionate
Unknow 2 (aromatic region)
Valerate
Lactose
Glucose
Pyruvate
3-hydroxyisovalerate
Succinate
Isobutyrate
Glutamate
N-acetylcysteine
Alanine
Site A
0,8 1,0 1,2 1,4 1,6 1,8 2,0 2,2 2,4 2,6 2,8 3,0
VIP scores

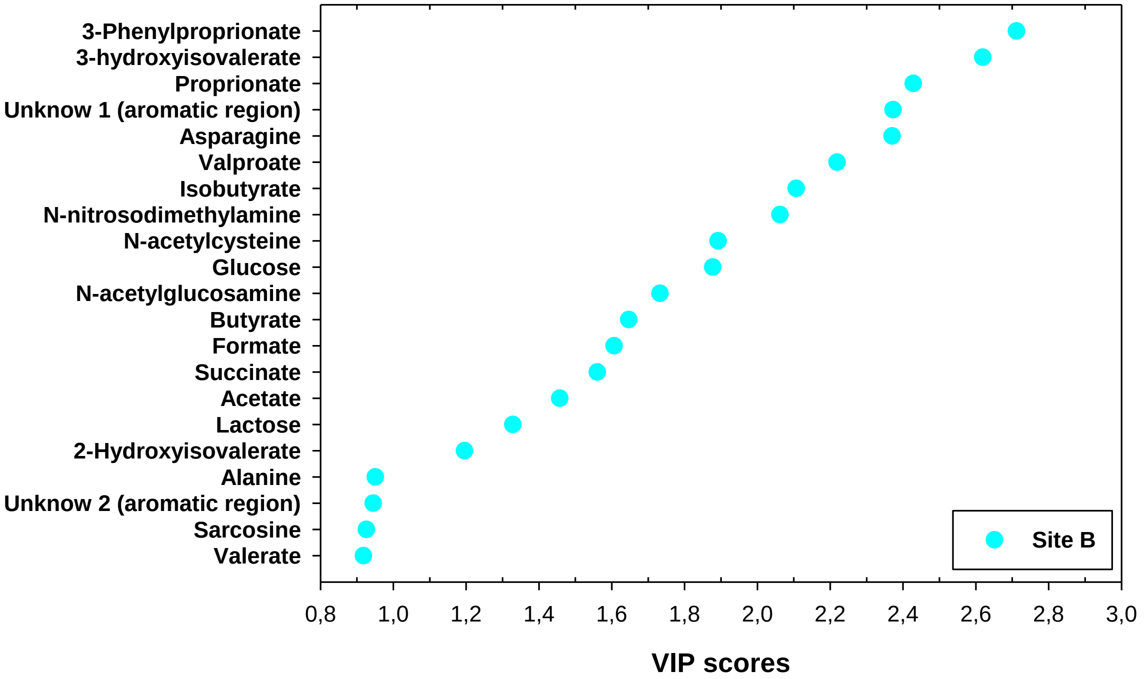
3-Phenylproprionate
3-hydroxyisovalerate
Proprionate
Unknow 1 (aromatic region)
Asparagine
Valproate
Isobutyrate
N-nitrosodimethylamine
N-acetylcysteine
Glucose
N-acetylglucosamine
Butyrate
Formate
Succinate
Acetate
Lactose
2-Hydroxyisovalerate
Alanine
Unknow 2 (aromatic region)
Sarcosine
Valerate
Site B
0,8 1,0 1,2 1,4 1,6 1,8 2,0 2,2 2,4 2,6 2,8 3,0
VIP scores

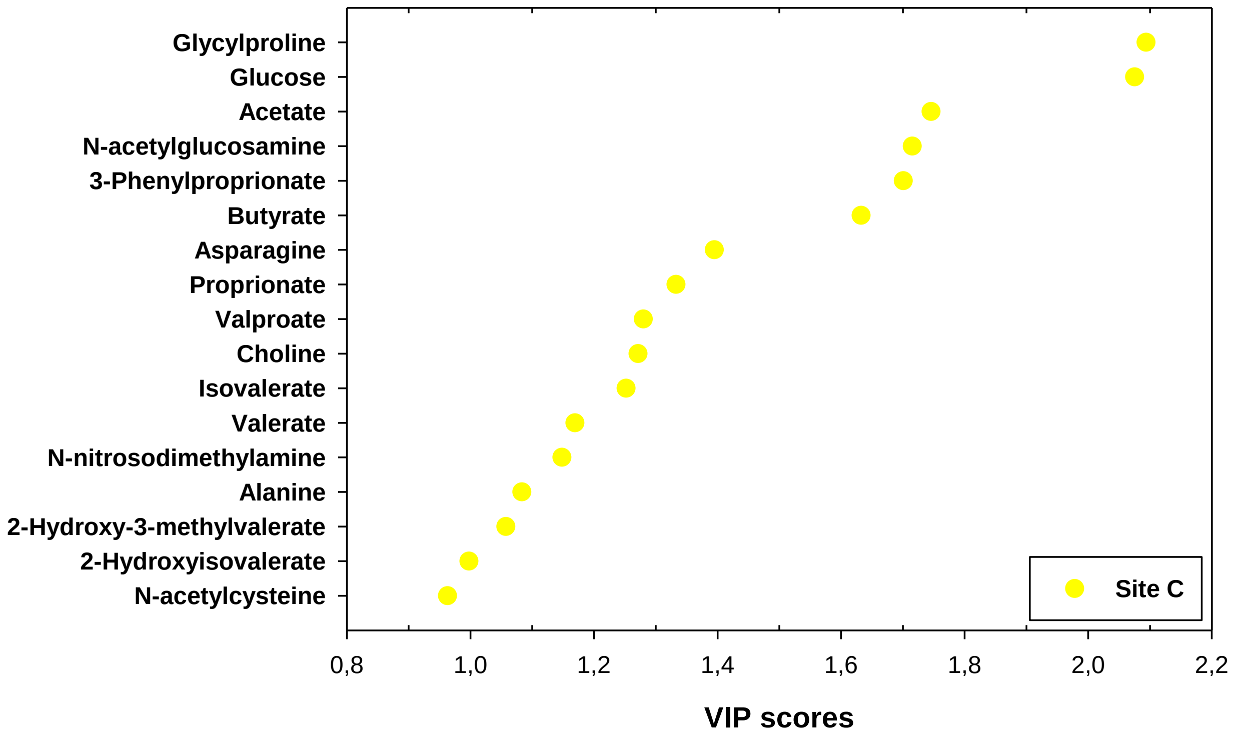

Figure S2. VIP score plots of the most important metabolites that contributed to the discrimination of the two cattle herds in low and high wolf-impacted areas within site A, B, and C. A total of 20, 21, and 17 metabolites, with a cut-off value of VIP > 0.9 and *P*-value < 0.05, were obtained from the OPLS-DA model of site A, B and C, respectively. For site A and B, where the interactions with wolves were higher compared to site C (Table 1), we identified 16 shared metabolites: 3-hydroxyisovalerate, 3-phenylproprionate, alanine, butyrate, glucose, isobutyrate, lactose, N-acetylcysteine, N-nitrosodimethylamine, proprionate, sarcosine, succinate, Unknown 1 (aromatic region), Unknown 2 (aromatic region), valerate and valproate. Of these 16 metabolites, nine were also found in site C: alanine, butyrate, glucose, N-acetylcysteine, N-nitrosodimethylamine, proprionate, 3-phenylproprionate, valerate and valproate.

Table S-1. Pearson's simple correlation between the PCA scores of cattle fecal extracts and distance to GPS radio-collared wolves for each site.

	PC1	PC2	PC3	PC4	PC5	PC6	PC7	PC8	PC9	PC10	PC11	PC12
**Site A**	0.10	**0.20**	0.13	0.10	0.16	0.15	0.07	**0.40**	0.28	-0.05	0.14	-0.03
**Site B**	-0.07	-0.07	**-0.26**	0.13	**-0.47**	0.00	0.23	**0.29**	-0.08	-	-	-
**Site C**	**0.30**	0.09	-0.17	**-0.40**	0.12	0.05	-	-	-	-	-	-

Absolute correlation values highlighted in bold were used as predictor variables for the regression analysis.
